# Implementing SBIRT (Screening, Brief Intervention and Referral to Treatment) in primary care: lessons learned from a multi-practice evaluation portfolio

**DOI:** 10.1186/s40985-017-0077-0

**Published:** 2017-12-29

**Authors:** Daniel Hargraves, Christopher White, Rachel Frederick, Margaret Cinibulk, Meriden Peters, Ashlee Young, Nancy Elder

**Affiliations:** 1 University of Cincinnati Department of Family and Community Medicine, Cincinnati, Ohio USA; 2 University of Cincinnati College of Medicine, Cincinnati, Ohio USA; 3 Interact For Health, Cincinnati, Ohio USA

**Keywords:** SBIRT (Screening, Brief Intervention, Referral to Treatment), Primary care, Substance abuse, Alcohol abuse

## Abstract

**Background:**

Screening, Brief Intervention and Referral to Treatment (SBIRT) is a public health framework approach used to identify and deliver services to those at risk for substance-use disorders, depression, and other mental health conditions. Primary care is the first entry to the healthcare system for many patients, and SBIRT offers potential to identify these patients early and assist in their treatment. There is a need for pragmatic “best practices” for implementing SBIRT in primary care offices geared toward frontline providers and office staff.

**Methods:**

Ten primary care practices were awarded small community grants to implement an SBIRT program in their location. Each practice chose the conditions for which they would screen, the screening tools, and how they would provide brief intervention and referral to treatment within their setting. An evaluation team communicated with each practice throughout the process, collecting quantitative and qualitative data regarding facilitators and barriers to SBIRT success. Using the editing method, the qualitative data were analyzed and key strategies for success are detailed for implementing SBIRT in primary care.

**Results:**

The SBIRT program practices included primary care offices, federally qualified health centers, school-based health centers, and a safety-net emergency department. Conditions screened for included alcohol abuse, drug abuse, depression, anxiety, child safety, and tobacco use. Across practices, 49,964 patients were eligible for screening and 36,394 pre-screens and 21,635 full screens were completed. From the qualitative data, eight best practices for primary care SBIRT are described: Have a practice champion; Utilize an interprofessional team; Define and communicate the details of each SBIRT step; Develop relationships with referral partners; Institute ongoing SBIRT training; Align SBIRT with the primary care office flow; Consider using a pre-screening instrument, when available; and Integrate SBIRT into the electronic health record.

**Conclusions and implications:**

SBIRT is an effective tool that can empower primary care providers to identify and treat patients with substance use and mental health problems before costly symptoms emerge. Using the pragmatic best practices we describe, primary care providers may improve their ability to successfully create, implement, and sustain SBIRT in their practices.

## Background

Substance use and mental health disorders are major global health issues. Worldwide, an estimated 240 million adults suffer from alcohol use disorder. Almost a quarter of adults use tobacco, which is responsible for approximately 11% of deaths in men and 6% of deaths in women [1]. The USA is currently experiencing an opioid epidemic, with catastrophic public health consequences. In 2015, the number of US drug overdose deaths rose to over 52,000 with 63% of these involving an opioid [2]. Meanwhile, depression presents one of the highest disease burdens worldwide [3]. Altogether, the disability-adjusted life-years due to mental and substance use disorders have increased by 15% from 2005 to 2015 [3]. These emerging data stress the need for sustainable, evidence-based public health initiatives that can reduce the impact of these conditions. Screening, Brief Intervention and Referral to Treatment (SBIRT) is a public health framework approach initially used to identify and deliver services to those at risk for the adverse consequences of alcohol abuse [4, 5], but which has been expanded to a number of substance-use disorders, depression, and other mental health conditions [4, 6].

Primary healthcare is key to preventing and finding disease early. However, in the USA, it has long been documented that there is insufficient time for all the preventive care needed [7]. SBIRT began in the 1960s as a screening and brief intervention tool to quickly identify those with risky alcohol use, saving time for providers by focusing on the highest need patients [5, 8].In the last several decades, research and demonstration projects (funded largely by the US Substance Abuse and Mental Health Services Administration (SAMHSA)) have confirmed that implementing SBIRT can positively impact patients and their communities [4, 9–12]. While not all research has yielded positive effects [3], the US Preventive Services Task Force (USPSTF) felt the evidence was strong enough to begin recommending screening and brief behavioral interventions for alcohol in 2004, and reaffirmed the recommendation in 2013 [13].

These demonstration projects have also recently begun assessing barriers and facilitators to successful SBIRT implementation [14, 15], the possibility of financial sustainability from clinical revenue [16, 17], and the effectiveness of various team members delivering SBIRT services [18]. Despite all this research, there is limited evidence for transferring this success from funded demonstration projects to day-to-day primary care office practice, or for beginning SBIRT screening in practices without significant external funding. Bernstein et al. describe lessons following a well-funded emergency department (ED) program, including external funding for start-up, local ED staff champions, sustainability planning from the beginning, and creation and maintenance of a robust referral network [15]. Singh et al. interviewed administrators and evaluators from six SAMHSA SBIRT grantee programs and found sustainability after the grant funding ended was related to securing new funding, having champions, adapting and making system changes, and managing program staffing challenges [17].Muench and Holland performed focus groups of team members and physicians in Oregon and Pennsylvania, respectively, during state-funded alcohol SBIRT projects [19, 20]. Both sets of researchers noted similar barriers, including time constraints, limited access to treatment, ongoing funding and reimbursement concerns, and limited knowledge and self-efficacy. While these studies provide a framework for primary care practices, they all come from large, well-funded projects where previously developed SBIRT was implemented in practices. While Dwinnels describes successful outcomes of a small SBIRT program in a regional community health center, he does not describe its sustainability, nor the factors associated with success [6].

Too many people today are not receiving the treatment they need for substance use and other mental health problems [21], and the growing opioid epidemic is a public health emergency [22]. Primary care is the entry to the healthcare system for the majority of patients across the globe. SBIRT offers great potential for primary care physicians and their staff to identify patients with risky substance use and early symptoms of mental illness and assist in their treatment. However, there is a need for pragmatic “best practices” for implementing SBIRT in primary care offices geared toward frontline providers and office staff. In 2014, The University of Cincinnati Department of Family and Community Medicine partnered with Interact for Health, a greater Cincinnati-based independent foundation, in evaluating SBIRT programs in 10 primary healthcare locations. From this work, we developed practical guidance for primary care practices to assist with developing and implementing SBIRT programs to help them address important public health issues in their communities.

### Basics of SBIRT

In the last 30 years, the SBIRT model has developed increasing function and utility. SAMHSA describes the three components of SBIRT as follows:

- Screening quickly assesses the severity of substance use and identifies the appropriate level of treatment.
- Brief intervention focuses on increasing insight and awareness regarding substance use and motivation toward behavioral change.
- Referral to treatment provides those identified as needing more extensive treatment with access to specialty care [23].

The SBIRT model has continued to grow due to its ability to be built on one of any validated screening instruments for a number of substance and mental health problems, be implemented in a variety of healthcare settings, be performed by a myriad of care team members, and be adapted for a number of culturally diverse populations [18, 24, 25]. For several conditions, “pre-screens” have been validated that allow for rapid, universal screening, followed by more focused full screens [26, 27].This has decreased the amount of time needed for screenings in primary care and other general populations. Because of the variety of conditions screened for, and the many settings where SBIRT can occur, there are no good population rates for its actual use, although a 2011 SAMHSA white paper did review the growing evidence for SBIRT’s effectiveness [25].

## Screening in primary care project

Between 2014 and 2016, Interact for Health awarded small grants (all US$60,000 or less) for the implementation of 10 SBIRT programs throughout the greater Cincinnati and Northern Kentucky region in an effort to reduce the number of people with risky substance use, anxiety, and depression. Unlike many previous SBIRT studies [19, 28, 29], each practice chose the condition or conditions for which they would screen, the screening tools, and how they would provide brief intervention and referral to treatment within their setting. An evaluation team from the University of Cincinnati’s Department of Family and Community Medicine (UC DFCM) communicated with each practice in an iterative process throughout the grant period and collected quantitative and qualitative data regarding facilitators and barriers to the SBIRT process.

### SBIRT practice descriptions

The SBIRT practices included primary care practices (family medicine and general internal medicine), federally qualified health centers (FQHCs), school-based health centers (SBHCs), and a safety-net emergency department (Table 1). Six of the practices screened for a single condition, while four practices screened for two to four conditions.

**Table 1:** Details about Screening, Brief Intervention and Referral to Treatment programs from the Interact for Health SBIRT Portfolio

	Condition(s) screened						Pre-screen	Full screen
	Alcohol	Substance use	Depression	Anxiety	Tobacco	Child safety		
Primary Care Prac. 1	●	●						AUDIT, DAST-10, CRAFFT
Primary Care Prac. 2			●				PHQ-2	PHQ-9
Primary Care Prac. 3						●		SEEK
Primary Care Prac. 4					●			Single current use question
Fed. Qual. Hlth. Cntr. 1	●						AUDIT-C	AUDIT
Fed. Qual. Hlth. Cntr 2	●	●	●				Single alcohol, drug question & PHQ-2	AUDIT, DAST-10, PHQ-9
Fed. Qual. Hlth. Cntr. 3	●						Single Alcohol Question	AUDIT-C
School-Based Cntr. 1	●	●	●					PHQ-9(A), CRAFFT
School-Based Cntr. 2	●							NIAAA
Emergency Department	●	●	●	●			PHQ-4	NM-ASSIST, PHQ-9, AUDIT, GAD-7

*AUDIT* Alcohol Use Disorders Identification Test, *NIAAA* National Institute on Alcohol Abuse and Alcoholism Screen for Youth, *PHQ-2, 4 or 9* Patient Health Questionnaire, *GAD-7* Generalized Anxiety Disorder 7, *DAST* the Drug Abuse Screening Test, *NM-ASSIST* National Institute on Drug Abuse Modified Alcohol, Smoking, and Substance Involvement Screening Test, *CRAFFT* Car-Relax-Alone-Forget-Friends-Trouble, *SEEK* Safe Environment for Every Kid

### Program evaluation methods

**Fig. 1:**
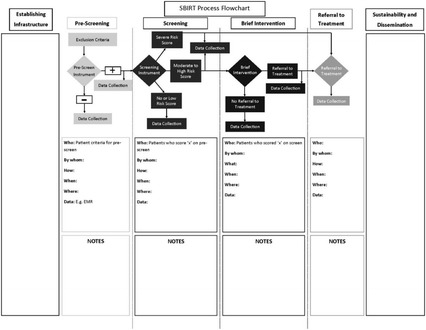
Graphical flow diagram of the SBIRT process used by practice leaders and staff for planning SBIRT implementation at primary care practices

Individual SBIRT programs varied in length from 9 to 18 months. The UC DFCM evaluation team met with each practice prior to the start of their program to help them develop process flowcharts that captured the corresponding action and personnel for each stage of SBIRT (Fig. 1). They then collected quarterly data via an online reporting system. Data collected included (1) number of patients eligible to be screened, (2) number screened, (3) number scoring positive on a screen, (4) number receiving a brief intervention, (5) number referred to treatment, and (6) number confirmed receiving treatment at referral location. In order to study real-time experiences of implementing and running SBIRT, qualitative data were also collected quarterly, both by brief interviews with evaluation staff and online open-ended questions. The evaluation team also visited most practices at least twice. Questions focused on what worked well during the previous quarter, what needed improvement, and what had changed in regard to data collection and/or SBIRT process flow.

Quantitative data were collated and summarized. Qualitative data, including open-ended question responses, practice visit notes, and interview notes, were collated and coded using the editing method [30, 31]. In this method, while acknowledging the existing literature about SBIRT in primary care [6, 12–14, 18, 19, 28, 32, 33], we sorted the interview data into coding categories derived from the data themselves, explicitly checking them against other categories and the original data, and then searched for patterns and themes. We then returned to the existing literature, and framed our findings as pragmatic best practices for successful implementation of SBIRT in primary care offices.

### Quantitative results

Across all ten program practices, an estimated 49,964 patients were eligible for screening. For all conditions, 36,394 pre-screens and 21,635 full screens were completed (19,687 adults and 1984 youth); 6203 scored positive on the full screens with 3108 brief interventions performed. Practices reported that 1302 referrals to treatment were made, but all practices reported an inability to confidently track confirmation of patients receiving treatment. Alcohol (7361) and substance use (7303) together comprised more than two thirds of the total full screens completed. Details of the SBIRT rates by conditions screened are found in Table 2.

**Table 2:** Numbers of patients receiving screening, brief intervention, and referral to treatment by type of condition

	Eligible for screening	Pre-screens completed	Positive pre-screens	Full screens completed	Positive full screens	Brief interventions^a^	Referrals to treatment^b^
Alcohol Abuse	22,360	12,697	2864	7361	1840	1009 (54.8%)	209 (20.7%)
Drug Abuse	16,419	5581	392	7303	1335	442 (33.1%)	172 (38.9%)
Adolescent Drug & Alcohol Abuse	9087	1868	421	794	55	91 (165.5%)	25 (27.5%)
Depression	23,861	14,062	3659	3706	2294	1050 (45.8%)	693 (66%)
Anxiety	2303	2186	537	74	10	8 (80%)	7 (87.5%)
Child Safety	1190	n/a	n/a	1057	251	193 (76.9%)	38 (19.7%)
Tobacco	1350	n/a	n/a	1340	418	315 (75.4%)	158 (50.2%)

^a^Percentage of positive full screens. Some practice sites administered a brief intervention regardless of the full screen score

^b^Percentage of brief interventions. Some practices bypassed brief interventions and referred some patients directly to treatment

*n/a* Not applicable as there were no pre-screening tools available

### Best practices for SBIRT implementation in primary care

#### Have a practice champion

This role is responsible for logistical coordination and problem-solving as well as provider accountability. The practice champion does not necessarily need to be the medical director of the practice, but should be someone who is respected by their coworkers. Several studies have cited the need for a champion to encourage staff buy-in and engagement and to identify and manage ongoing barriers to program success [20, 34]. This was consistent with our findings where a program leader who could act as cheerleader, door opener, and bridge between all team members was key to a successfully integrated program. Practice champions who are not in leadership positions need the support and backing of leadership. When the program leader was not a clinical leader, practices that included the medical or nursing director in planning meetings and decision making were more likely to have earlier success. With increasing and competing demands in healthcare settings nationwide, it is necessary to have a point person capable of securing buy-in from the necessary care team members, obtaining initial resources, and ensuring judicious use resources as the program continues.

#### Utilize an interprofessional team

Incorporating physicians, medical assistants, information technology staff, front desk staff, and other essential staff can aid in identifying challenges and optimizing the process for maximum patient impact. Physicians often mention their lack of time as a major barrier to SBIRT [14, 19]. Involving an interprofessional team can mitigate the physician’s role in favor of shared responsibility among all participants in the SBIRT continuum of care [18, 20, 28]. These interprofessional team members need to be involved from the planning stage. Several of our practices failed to include all team members in the planning process. This resulted in disjointed program rollouts at these practices with wasted resources and the need for additional time and energy to make major midcourse corrections. Coordination and communication across disciplines and between diverse skillsets is necessary for seamless and complete delivery of all SBIRT stages.

#### Define and communicate within the team details of each SBIRT step

Each SBIRT component should be determined based on the needs and availability at the primary care practice as well as provider interest and experience [14, 18, 20, 35]. Early identification of the conditions to be screened and selection of appropriate and validated tools is the first, but essential step, as it will focus and guide the rest of the process. However, our participants found that creating, implementing, and documenting the brief intervention was actually one of the most difficult parts of implementing SBIRT. Practices that created detailed brief intervention expectations (who, when, where, how long, and how often) had more successful outcomes. We also found that practices screening for multiple conditions were unable to offer brief interventions or referrals for multiple positive screens due to time and staff availability. These practices created algorithms that prioritized one positive screen (e.g., drug abuse) over another (e.g., depression) for brief intervention. A limitation of these algorithms is that they were operationalized by screening staff who had limited clinical training and thus were not always patient centered. Primary care practices should consider patient survey fatigue, as well as their own capacity to offer interventions and referrals in a timely manner should they decide to screen for multiple conditions.

#### Develop relationships with referral partners

All practices failed to implement a referral to treatment that included a communication feedback loop to primary care. Adequate referral partner relationships are necessary for high-risk patients. To better link patients with treatment options after a positive screen and brief intervention, referral partners should be brought to the table during the planning phase of SBIRT. Additionally, other options such as telephonic or telehealth treatment should be explored to increase access to treatment as part of SBIRT [34]. In our region, the lack of referral resources, especially those that can accept a variety of healthcare insurance, were noted as a significant weakness of the implemented SBIRT programs. Additionally, lack of feedback from referral centers made tracking difficult. The confidentiality that is afforded to mental health and substance abuse records further complicated this process. An open line of communication between referring and referral partners and inclusion during SBIRT planning can help to mediate follow-up barriers, thereby ensuring timely and accurate feedback on treatment linkages. Integrated practices incorporating mental health and/or substance abuse care with primary care also show promise as method for improving both care and communication [36].

#### Institute ongoing SBIRT training

Because primary care SBIRT relies on an interprofessional team, training of all involved parties is integral to program success. Staff turnover and insufficient training have been cited as barriers to SBIRT success [18, 20, 34] and full program implementation may require up to 12 months [18] with continued training and education. As with many primary care offices, our practices were vulnerable to staff turnover. Keeping this in mind, training protocols should be a part of the original planning and program design. SBIRT training should also be incorporated into the onboarding process to maximize success through any staff transition by building broad institutional memory.

#### Align SBIRT within the primary care office flow

As part of the planning phase, a graphical flow alignment diagram that follows the patient through the SBIRT process from beginning to end is useful in assuring that SBIRT fits within existing office flow, such as outlined in Fig. 1. Specifically, flow diagrams that clearly define the pre-screen and screening instrument to be used, scores that lead to brief intervention or directly to treatment, and identify the staff responsible for each step help create an SBIRT program that can more seamlessly integrate into the practice. A graphical flow diagram allows for process refinement prior to implementation. Data collection processes should be included in the operational plan, as feedback is necessary to assure that SBIRT outcomes are being met. Universally, our practices that created, communicated, adapted, and revised the flow diagrams during the planning phase had fewer problems as they rolled out their SBIRT programs. These formal visual maps minimized potential problems before they arose by defining the team and assigning ownership of various SBIRT components.

#### Consider using a pre-screening instrument, when available

A major concern by primary care staff who perform the screening of SBIRT is time [20]. Using brief, validated pre-screens can decrease the amount of time spent administering longer instruments, and increase the yield from the full screens. For example, two FQHC practices screened for alcohol abuse, one using the full Alcohol Use Disorder Identification Test (AUDIT) for everyone and one using the AUDIT-C pre-screen, followed by the full AUDIT for those with positive pre-screens. The center using only the full AUDIT had a 5% rate of positive screens, but everyone had to complete the full AUDIT. The center using the pre-screen had 30% of their patients pre-screen positive, so only this smaller number completed the full AUDIT and 74% of them had positive full screens. Incorporation of pre-screening into mature SBIRT programs has been utilized to address concerns regarding sustainability and ensure judicious use of staff time while increasing the number of patients served [34]. Whenever possible, validated pre-screening instruments should be utilized.

#### Integrate SBIRT into the electronic health record (EHR)

The ability to track patients through the SBIRT process via the EHR is necessary for documenting patient care, analysis of program impact, and assisting practices with population health by better defining and managing the patient population identified by SBIRT. Applicable coding ensures more accurate billing and allows for potential reimbursement for the screening and brief intervention which has been noted as a necessity for program sustainability [15, 16]. Additionally, other EHR tools such as automated reminders increased the number of patients screened at our program practices. The EHR needs to clearly flag or highlight positive screens to ensure that brief interventions are delivered [37]. Attention must be paid to the EHR integration during the planning phase, however, or lost revenue and poor outcome documentation can sink a program before it becomes established.

## Discussion

As initial programmatic and research funding for SBIRT ended, significant questions still remained about how to create and maintain sustainable SBIRT programs in primary care settings. With the USPSTF supporting the regular use of SBIRT for alcohol abuse [13], and strong evidence for SBIRT growing for other conditions [6, 9–12], primary care offices need practical guidance for how best to create and implement SBIRT programs. Since the literature has done a better job of describing barriers to SBIRT than facilitators [5, 10, 14, 16, 17, 19, 20, 24, 34, 38], we took the lessons learned from our qualitative evaluation of 10 diverse practices and created 8 pragmatic best practices. Many of these are further evidence supporting existing recommendations. For example, the need for practice champions, creating a robust referral network, planning for sustainability, and using an interprofessional team have been described in the SBIRT literature [15, 17, 18, 28]. We have added, however, specific details gleaned from working with practices that created SBIRT programs internally, with minimal external funding, in order to provide guidance for primary care physicians, staff and administrators interested in implementing their own SBIRT program.

There are limitations to our study. The 10 practices were selected through a competitive grant process by the community agency, Interact for Health, and therefore might be different than other practices in the community. The greater Cincinnati-Northern Kentucky region is a mid-sized metropolitan region in the midwest USA and is likely different in primary care and clinical practice than other locations in the country. And while the program was created for screening in primary care, the funder included practices such as a safety-net emergency department that many would not consider a primary care location. However, most of the practices were family medicine or general internal medicine offices, school-based clinics, or community health centers. The qualitative findings were consistent with findings from the medical literature [15, 17, 18, 20] making it likely that the practical best practices from these practices will be of value to primary care practices seeking to implement SBIRT.

## Conclusion

The sustainability of an SBIRT program in a primary care setting relies heavily on a well-defined and operationalized plan that fits within office flow. Having a practice champion as well as bringing key members of the team on board in the planning stages improves the chances of successful implementation and continued SBIRT delivery. With our current opioid epidemic, perhaps more than any other time in recent history, primary care must take action and fully participate in identifying patients at risk of substance use and mental health problems. In addition to current community-based prevention programs, public health models like SBIRT in primary care are needed to make a concerted effort against the downstream effects of substance use and mental illness. SBIRT has been shown to be an effective tool that can empower primary care providers to identify and treat this population before costly symptoms emerge. Using the pragmatic best practices we describe, primary care practices may improve their ability to successfully create, implement, and sustain SBIRT programs.

## Abbreviations

AUDIT: Alcohol Use Disorders Identification Test
CRAFFT: Car-Relax-Alone-Forget-Friends-Trouble
DAST: Drug Abuse Screening Test
ED: Emergency department
EHR: Electronic health record
FQHC: Federally qualified health center
GAD: Generalized anxiety disorder
NIAAA: National Institute on Alcohol Abuse and Alcoholism Screen for Youth
NM-ASSIST: National Institute on Drug Abuse Modified Alcohol, Smoking, and Substance Involvement Screening Test
PHQ: Patient Health Questionnaire
SAMHSA: Substance Abuse and Mental Health Services Administration
SBIRT: Screening, Brief Intervention and Referral to Treatment
SEEK: Safe Environment for Every Kid
UC DFCM: University of Cincinnati’s Department of Family and Community Medicine
US: United States
USPSTF: United States Preventive Services Task Force
